# Supplementary material for: Impact of Covid-19 control strategies on health and GDP growth outcomes in 193 sovereign jurisdictions
Source: PLOS Glob Public Health. 2025 Oct 16;5(10):e0004554. doi: 10.1371/journal.pgph.0004554 (PMC12530520; doi:10.1371/journal.pgph.0004554)
Supplement: Attachment — Submitted filename: 250714 Response to Reviewers.docx [file pgph.0004554.s002.docx]

**Impact of Covid-19 Control Strategies on Health and GDP Growth Outcomes in 193 Sovereign Jurisdictions**

**PGPH-D-25-00821**

**Response to Reviewers**

**Response to Editor**

**Journal Requirements:**
1. “Fig 1.tiff and Fig 2.tiff” files are over our file size limit of 10MB. This limit is in place for the convenience of reviewers, editors and readers. Please adapt this file so that the file size is below 10MB. You may find it helpful to consult our guidelines on compressing figures here: <https://journals.plos.org/globalpublichealth/s/figures>

**Authors’ response:** Thank you, we have now processed the Figures using the NAAS tool as suggested on the PLOS website: <https://journals.plos.org/globalpublichealth/s/figures>

Both figures are now less than 10MB.

**Data Repository:**

3. Have the authors made all data underlying the findings in their manuscript fully available (please refer to the Data Availability Statement at the start of the manuscript PDF file)?

The [PLOS Data policy](https://track.editorialmanager.com/CL0/https:%2F%2Fjournals.plos.org%2Fglobalpublichealth%2Fs%2Fdata-availability/1/010f019777bc917b-61378ff1-4b4d-4ca5-8950-d12843176aae-000000/nQr9_ppVzrbz0WST2Gc13BLP-rEXtlicp1nM6WsUZ4A=215) requires authors to make all data underlying the findings described in their manuscript fully available without restriction, with rare exception. The data should be provided as part of the manuscript or its supporting information, or deposited to a public repository. For example, in addition to summary statistics, the data points behind means, medians and variance measures should be available. If there are restrictions on publicly sharing data—e.g. participant privacy or use of data from a third party—those must be specified.

Reviewer #1: Yes

Reviewer #2: No

**Authors’ response:** Data is available at this link (which we have added to the Data Availability section on p.46): <https://adaptresearch.wordpress.com/wp-content/uploads/2025/07/250317-updated-master-uploaded-to-adapt-site.xls>

**Response to Peer Reviewers**

Reviewer #1: Overall comment:
Please include line numbers to easily reference issues in the paper next time.

The authors intended to evaluate the impact of Covid-19 control strategies on Health and GDP growth outcomes in 193 sovereign jurisdictions. The strengths of this paper is in the statistical analysis. It is well stipulated. The weakness of the paper is that you assume that everybody knows what you are talking about and have not defined the major terms in your study. You do not mention the elimination methods too.

**Authors’ response:** Thank you for this constructive feedback about improving our definitions and helping readers. We have now addressed the individual specific issues you raise, see below.

Detailed comments:
How would you define age standardized excess mortality? How would you define: GDP per capita, GDP growth.

**Authors’ response:** We have added further clarity around several of the definitions. Specifically:

We have added the following text on p.14, “the age-standardized excess mortality variable represents the difference between observed all-cause deaths and expected deaths (based on pre-pandemic trends) during 2020-2021, standardized across age groups to enable comparison between populations with different age structures.”

Also the following on p.14, “The World Bank defines GDP per capita as GDP divided by midyear population. Specifically, we obtained the World Bank GDP per capita purchasing power parity (PPP) (constant 2017 international $) data, which included adjustment for inflation, ie, the World Bank dataset “NY.GDP.PCAP.PP.KD”. We then calculated GDP growth from 2019 to 2020, and from 2020 to 2021, defined as the percentage change in five-year geometric means. In some analyses we report results for ‘Low’, ‘Medium’ and ‘High’ GDP jurisdictions based on tertiles of 2019 GDP.”

Abstract: Looks good
Introduction: Looks good
Hypotheses: Look good

Methods and materials:
“We defined islands as per previous work” –we are reading this one and not your previous work. Please define it again and then you can reference your previous work.

**Authors’ response:** We have now added the following specific definition (p.9) (consistent with how we defined islands in our previous work), “We defined island jurisdictions in the dataset as those surrounded by water, while ignoring structural connections to other land masses such as bridges, causeways and tunnels (and so including Singapore, Bahrain and the UK as islands). In summary, the group of island jurisdictions included the following: individual sovereign islands, island archipelagos (eg, Indonesia, Philippines), island continents (ie, Australia), and where the jurisdiction had a land border with another jurisdiction on the same island (eg, Ireland, UK, Timor-Leste, Papua New Guinea, Brunei, Dominican Republic, Haiti, Cyprus). We excluded South Korea (although it can be considered a pseudo-island) and jurisdictions with mixed characteristics but where the capital city was on a continental land mass (eg, Malaysia, Corsica [France], Sardinia and Sicily [Italy]).”

Excess mortality data: Please mention the elimination methods used/ considered in this study.

**Authors’ response:** We have now added a table, designated Table 1, which identifies what the 5 ‘explicit elimination’ jurisdictions actually implemented.

Results: Well explained

Like I said earlier, we are working blindly with your terms. Define Low GDP, Middle and high GDP. What parameters were you looking at to define corruption in your jurisdictions for this study?

**Authors’ response:** We defined Low, Medium, and High GDP based on tertiles of 2019 GDP (as indicated in the footnotes to Table 5 where the Low/Medium/High results appear). We have also now included the following statement in the Methods: “In some analyses we report results for ‘Low’, ‘Medium’ and ‘High’ GDP jurisdictions based on tertiles of 2019 GDP.” (p.15)

Discussion:
“Our analysis revealed several key patterns regarding the relationship between Covid- 19 control strategies, border restrictions, and health outcomes” – Is GDP a health outcome? Rephrase.

**Authors’ response:** We have rephrased this to read ‘health and economic’ outcomes.

Reviewer #2: I should state that I am not a subject matter expert on this manuscript, I was able to read through with some observations:

1. The authors were able to comprehensively deal with the subject matter and provided references (sources) from which the data were extracted. However, there were no minimum data sets submitted.

**Authors’ response:** Here is a link to the data: <https://adaptresearch.wordpress.com/wp-content/uploads/2025/07/250317-updated-master-uploaded-to-adapt-site.xls>

2. Going by the calculations in Table 1 under the results, there appears to be some inconsistencies in the representation of percentages of the figures stated under ''Number reaching level 4 border control''. For instance, under All jurisdictions, a percentage of 82.8% was given which, i think should be 82.4% (82.38%); Non-island jurisdictions given as 83.3% ought to be 82.8% (82.75%) ; Island jurisdictions 81.2% ought to be 81.3% (81.25%) and Non-exclusion/elimination jurisdictions 83.4% ought to be 83% (82.97%). The same observations were made under super-region, Sub-Saharan Africa 97.9% which ought to be 95.8% Under region, Caribbean given as 81.2% ought to be 81.3% (81.25%) while Central SubSaharan Africa given as 100% ought to be 83.3%. It is not clear how these figures will affect other tables.

**Authors’ response:** Thank you for spotting this. This problem occurred because one jurisdiction (Equatorial Guinea) did not have data in the OWID dataset for whether or not it achieved Level 4, so reading across the row it was included in age-standardized EM denominator (where data existed), but should not be included in the denominator for ‘reaching’ level 4. We have amended the second column to indicate instances where the denominator is different due to this data gap. This data gap fully explains the discrepancies the Reviewer thoughtfully observed.

The two instances of 81.2 that should be 82.3 were both cases of truncation of “82.25” in the readout rather than upward rounding to 82.3 and have been corrected.

Other tables are not affected as N is reported individually for each analysis, and % are not calculated, as the results are regression coefficients etc.

**Authors’ Final Comment:** We thank the Editor and the Reviewers for their very helpful feedback and the opportunity to revise this manuscript. It is undoubtedly strengthened by the suggestions and corresponding revisions.
